# Supplementary material for: Role of Autophagy-Related Gene atg22 in Developmental Process and Virulence of Fusarium oxysporum
Source: Genes (Basel). 2019 May 13;10(5):365. doi: 10.3390/genes10050365 (PMC6562804; doi:10.3390/genes10050365)
Supplement: Supplementary file 1 [file genes-10-00365-s001.pdf]

## Supplementary Materials

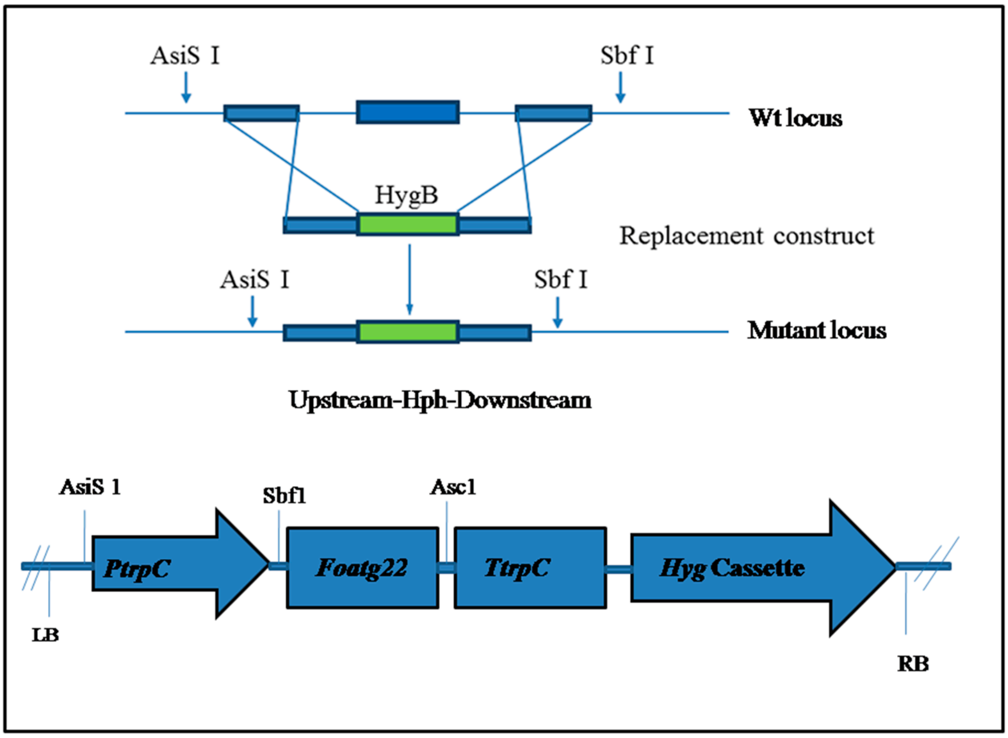


**Figure S1.** Construction of deletion and overexpressed mutants. (A) The *Foatg22* coding region was replaced with the HygB cassette. (B) The *Foatg22* cDNA fragment was amplified with the indicated primers containing Sbf1 and Asc1 restriction sites inserted at 5′ and 3′ end respectively.

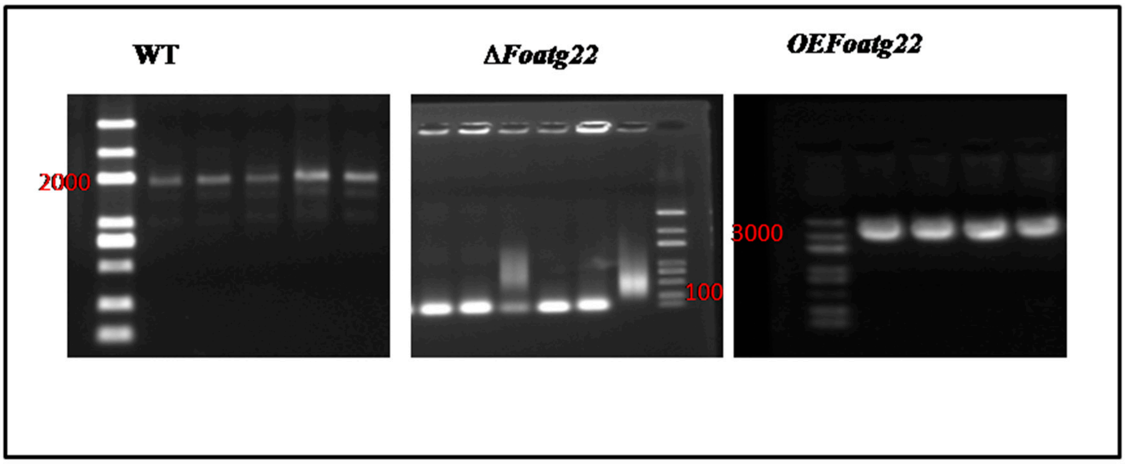


**Figure S2.** Molecular identification of mutants. Image represents the expected shift in deletion (Δ) and over expression of mutants. The arrows indicate the target bands.

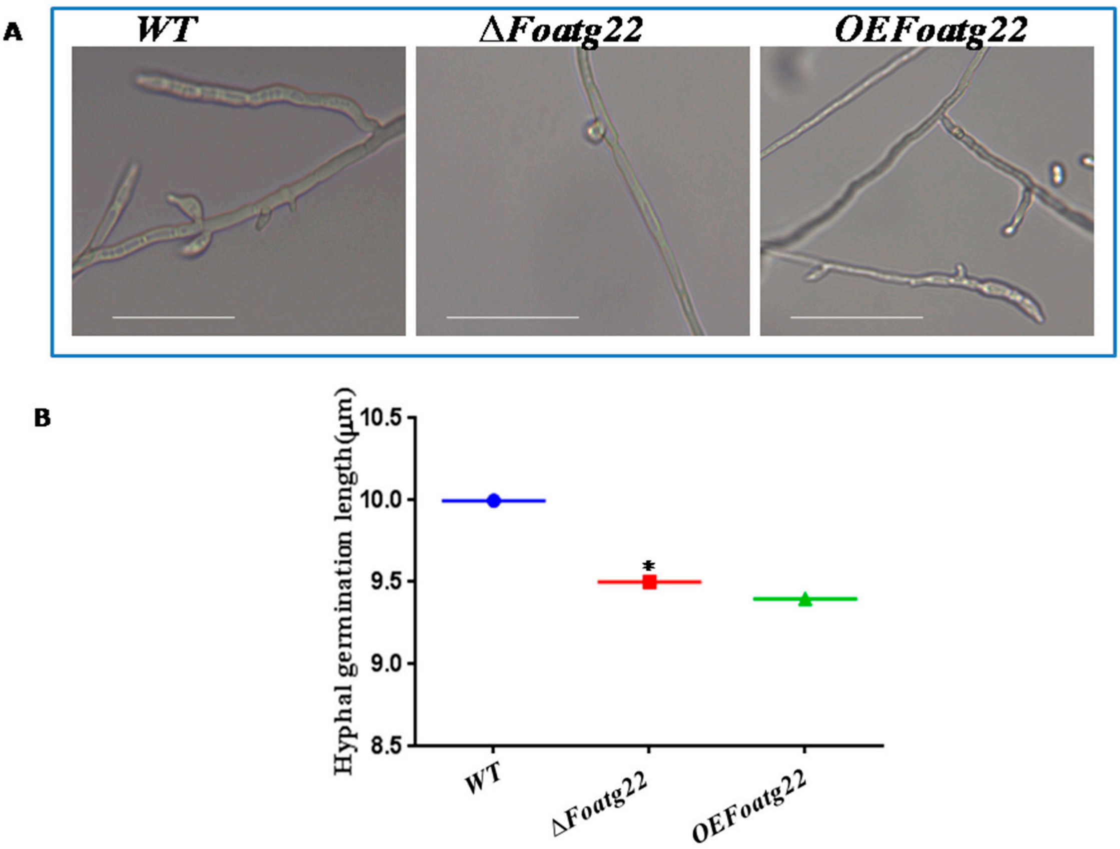


**Figure S3.** Representative hyphal growth and length of WT, *ΔFoatg22*, Overexpression of *Foatg22*. (A) Indicating the hyphal germination density. (B) Hyphal germination length. (Duncan, $P < 0.05$). Bars= 10μm.

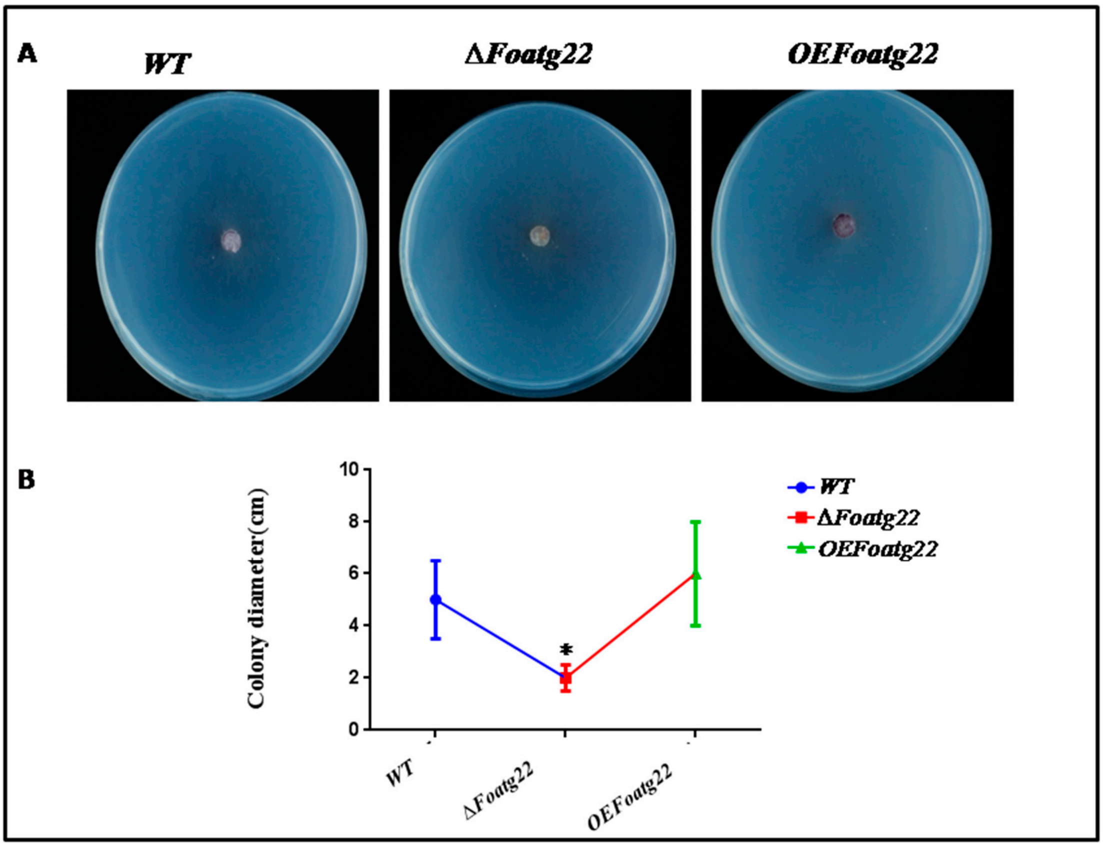

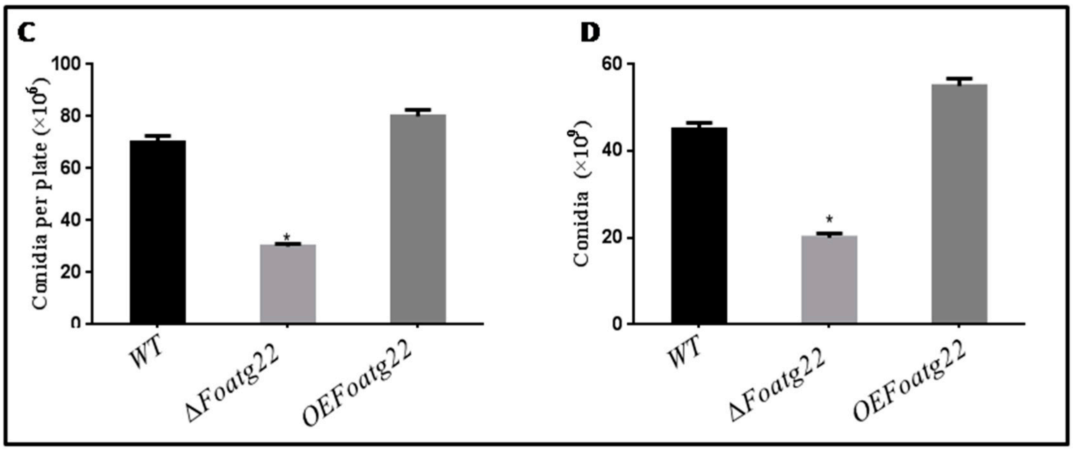


**Figure S4.** Representative phenotype of WT, *ΔFoatg22* and Overexpression of *Foatg22*. In Δ*FoATG22* mutant conidial formation and hyphal formation was reduced. (**A**) Represents the image of 14 days old strains after inoculation. (**B)** Freshly micro conidia ($10^3$) were inoculated in to PDA plates and then incubated at 28°C. Colony diameter was measured on daily basis till ten days. Graph represents growth rate of strains. Conidial formation was significantly reduced in *ΔFoatg22* (**C**) After 14 days number of micro conidia recovered from PDA plates which were grown on 28°C. (**D**) Represents the number of recovered micro conidia from 2 days old culture with shaking at 28°C. Conidial formation was reduced significantly in *ΔFoatg22*.Bars indicating slandered error from replications. To perform statistical analysis Duncan post-hoc test was used (Duncan, $P < 0.05$).

**Table S1.** Primers used in present study**.** Primers used for construction of replacement cassette and genes cloning.

| Gene name | Primer name | Primer sequence (5` to 3`) | T ° |
|---|---|---|---|
| | | **Deletion primers** | |
| **Foatg22** | FoATG22 5′-U F (P1) | GGTACCTGCGTTAACTCACTGAGAAGTAAGCAGAA | 80 |
| | FoATG22 5′-U R (P2) | GTTCAGGCTTTTTCATGGTGGCGGCCGCGGCTACAG GTAGCTATGTCG | 89.7 |
| | FoATG22-Hph-F(P3) | CGACATAGCTACCTGTAGCCGCGGCCGCCACCATG AAAAAGCCTGAAC | 89.7 |
| | FoATG22-Hph-R (P4) | AATCAACAGCCATCTGAGCCTGCAGGCTATTCCTTT GCCCTCGG | 87.4 |
| | FoATG22 3′-D F (P5) | CCGAGGGCAAAGGAATAGCCTGCAGGCTCAGATGG CTGTTGATT | 81.3 |
| | FoATG22 3′-D R (P6) | GGATCCCGATCTCTTGCGGTTGCTCATTGTG | |
| | | **Cloning primers** | |
| **Foatg22** | FoATG22 F | CCTGCAGG ATGGCGCCAAACCTTCAACCTC | 62.6 |
| | FoATG22 R | GGCGCGCC GGGTCTTGCGTGTTCATCGACA | 61.8 |
| | PtrpC F | TTAGCAGACAGGAACGAGGACAT | 56.7 |
| | | **qRT-PCR** | |
| **Foatg22** | RT FoAtg22F | CTCACGGCTCTTTAACCTCC | 54.5 |
| | RT FoAtg22R | TATACAGCCGCCAGTGGATA | 54.5 |
| | RT FOXG_04522F1 | TCTCTACGGCACTTCTCAAT | 55.6 |
| | RT FOXG_04522R1 | TTGGTCAAGAATAGGCAGGA | 55.6 |
